# The current profile of persistent cloaca and cloacal exstrophy in Japan: the results of a nationwide survey in 2014 and a review of the literature

**DOI:** 10.1007/s00383-016-4053-4

**Published:** 2017-01-05

**Authors:** Masayuki Kubota

**Affiliations:** Department of Pediatric Surgery, Niigata University Graduate School of Medical and Dental Sciences, 1-757 Asahimachi-Dori, Chuo-ku, Niigata, 951–8510 Japan

**Keywords:** Persistent cloaca, Cloacal exstrophy, A nationwide survey, Menstrual outflow obstruction

## Abstract

**Purpose:**

The current profile of persistent cloaca (PC) and cloacal exstrophy (CE) in Japan was first examined in 2014.

**Materials and methods:**

Information was obtained by sending a questionnaire to 244 university hospitals and children’s hospitals.

**Results:**

Responses from 113 institutions reported 466 PC cases and 229 CE cases. The incidences of PC and CE from 1980 to 2012 were 0.97 and 0.49 per 100,000 live births, respectively. In the previous 5 years, antenatal abnormalities were found in 57.6% of PC and 72.7% of CE patients. Myelomeningocele was observed in 45.6% of CE patients. As a result of various surgical treatments used in the neonatal and infantile periods, the respective rates of bladder dysfunction, clean intermittent catheterization, and permanent enterostomy were 32.6, 22.5, and 7.3% in PC patients and 60.7, 28.4, and 73.8% in CE patients. Menstrual outflow obstruction was found in 22.5% of PC and 48.9% of CE patients with menstruation.

**Conclusion:**

The clinical outcomes of PC and CE remain unsatisfactory. Therefore, the establishment of treatment guidelines might be a useful objective for improving the current status of PC and CE.

## Introduction

Persistent cloaca (PC) and cloacal exstrophy (CE) are extremely rare congenital anomalies of the anorectum and urogenital tract. PC occurs exclusively in female patients, where the rectum, vagina and urethra converge into a common channel, resulting in a single perineal opening. In contrast, CE is the most severe form of cloaca-related anomaly and it occurs in both sexes; it is characterized by a failure of lower abdominal wall closure, resulting in exstrophy of the intestines and urinary and genital organs. These two diseases are usually associated with multiple anomalies of other organs, which also affect the treatment outcome considerably. Carey et al. [1] described a combination of defects, consisting of omphalocele, exstrophy of the cloaca, imperforate anus, and spinal defects as “OEIS complex”.

Because the functional outcomes of surgical treatment of the reproductive system in infancy will become obvious in puberty, these patients need meticulous care from childhood to adulthood. To perform suitable life-long care in these patients, treatment guidelines must be established. However, the precise details of these issues are largely unclear. Furthermore, basic information regarding PC and CE in the perinatal period, such as the incidence, rate of antenatal diagnosis, and mode of delivery, are also unavailable, due to the lack of a nationwide survey of these two diseases.

We therefore conducted a nationwide survey of PC and CE in 2014 to determine the current status of these diseases in Japan before establishing treatment guidelines. To our knowledge, this survey is the first nationwide survey of PC and CE, performed simultaneously. The results of our survey are introduced here with a review of the literature.

## Patients and methods

This research project of general-068 entitled, “An establishment of treatment guidelines for smooth transitional care of persistent cloaca, cloacal exstrophy and Mayer-Rokitansky-Kuster-Hauser syndrome,” was supported by the Health and Labour Sciences Research Grants for Research on Rare and Intractable Diseases from 2014 to 2016 in Japan. The results of a nationwide survey concerning PC and CE are presented in this manuscript. Table 1 lists the research members who participated in the present survey. The Ethics Committee of Niigata University School of Medicine approved the study protocol of the research project and nationwide survey (approved number of 1888).

**Table 1:** Lists of research members participating in the nationwide survey

Name	Institute, department
Yutaka OSUGA	Graduate School of Medicine, the University of Tokyo, Department of Obstetrics and Gynecology
Kiyoko KATO	Graduate School of Medial Sciences Kyushu University, Department of Gynecology and Obstetrics
Kenji ISHIKURA	National Center for Child Health and Development, Division of Nephrology and Rheumatology
Kazunari KANEKO	Kansai Medical University, Department of Pediatrics
Kohei AKAZAWA	Niigata University Graduate School of Medical and Dental Sciences, Department of Medical Informatics
Yoshiaki KINOSHITA	Graduate School of Medical Sciences Kyushu University, Department of Pediatric Surgery
Takeo YONEKURA	Nara Hospital, Kindai University Faculty of Medicine, Department of Pediatric Surgery
Yuko TAZUKE	Osaka University Graduate School of Medicine, Department of Pediatric Surgery
Satoshi IEIRI	Kagoshima University, Research Field in Medicine and Health Sciences, Department of Pediatric Surgery
Akihiro FUJINO	National Center for Child Health and Development, Devision of Pediatric Surgery
Shigeru UENO	Tokai University School of Medicine, Department of Pediatric Surgery
Yutaro HAYASHI	Nagoya City University Graduate School of Medical Sciences, Department of Nephro-urology
Kaoru YOSHINO	Aichi Children’s Health and Medical Center, Department of Urology
Toshihiro YANAI	Ibaraki Children’s Hospital, Department of Pediatric Surgery and Pediatric Urology
Jun IWAI	Chiba Children’s Hospital, Department of Pediatric Surgery
Takanori YAMAGUCHI	Fukuoka Children’s Hospital, Department of Urology
Shintaro AMAE	Ekoh-Ryoikuen Hospital-homes for persons with severe motor and intellectual disabilities (SMID), Department of Surgery
Yuichiro YAMAZAKI	Kanagawa Children’s Medical Center, Department of Urology
Yoshifumi SUGITA	Kobe Children’s Hospital, Department of Urology
Miyuki KOHNO	Kanazawa Medical University, Department of Pediatric Surgery
Yutaka KANAMORI	National Center for Child Health and Development, Devision of Pediatric Surgery
Yuko BITOH	Kobe University Hospital, Department of Pediatric Surgery
Masato SHINKAI	Kanagawa Children’s Medical Center, Department of Surgery
Yasuharu OHNO	Oita University Faculty of Medicine, Department of Gastroenterological and Pediatric Surgery
Yuhki ARAI	Niigata University Graduate School of Medical and Dental Sciences, Department of Pediatric Surgery

Information was obtained by sending a questionnaire to 244 university hospitals and children’s hospitals in Japan to investigate the etiologic events and clinical outcomes in patients with PC and CE. Responses were obtained from 113 institutions (46.3%) regarding 466 cases of PC and 229 cases of CE, which were the total of cases treated in the past. Roughly 300 events and parameters were examined in the following order: perinatal events, associated anomalies, surgical treatments shortly after birth, radical operations, renal and bladder function, reproductive organ function, and school and social life. Since national birth statistics in Japan were available up to 2012, the incidence of diseases for every 100,000 live births was calculated for patients born between 1980 and 2012.

## Statistical analyses

The statistical analyses were performed using a commercially available software program (Prism 6 for Mac OS X version 6.0b; La Jolla, California, USA). The results are expressed as the median. Statistically significant differences between the groups were estimated using independent Student’s *t* test or the Mann-Whitney *U* test. Differences were considered to be significant for values of *p* < 0.05.

## Embryology

PC and CE are considered to be a spectrum of disease that affects the formation of the cloaca. CE patients show the most severe form of cloacal anomaly, associated with omphalocele, pubic bone dehiscence, and frequently with myelomeningocele. Briefly, the body wall is constructed in the fourth gestational week. In the same period, the neural tube is also created. Major events that cause the failure of the anterior abdominal wall closure can therefore also disturb the neural tube formation, which might be a reason for the strong association with myelomenigocele in CE patients [2]. The division of the cloaca occurs in the next developmental stage. The urorectal septum differentiates the cloaca into the urogenital tract and the anorectal tract [3, 4]. However, a recent anatomical study using animal models of anorectal malformation found that the normally developing cloaca differed starkly from any form of anorectal malformation, including persistent cloaca, where the cloacal membrane was too short to function as the dorsal cloaca [5]. Therefore, further studies are necessary to clarify the precise developmental aspect of PC and CE.

## Results

### Perinatal events

#### Prevalence of PC and CE

**Fig. 1:**
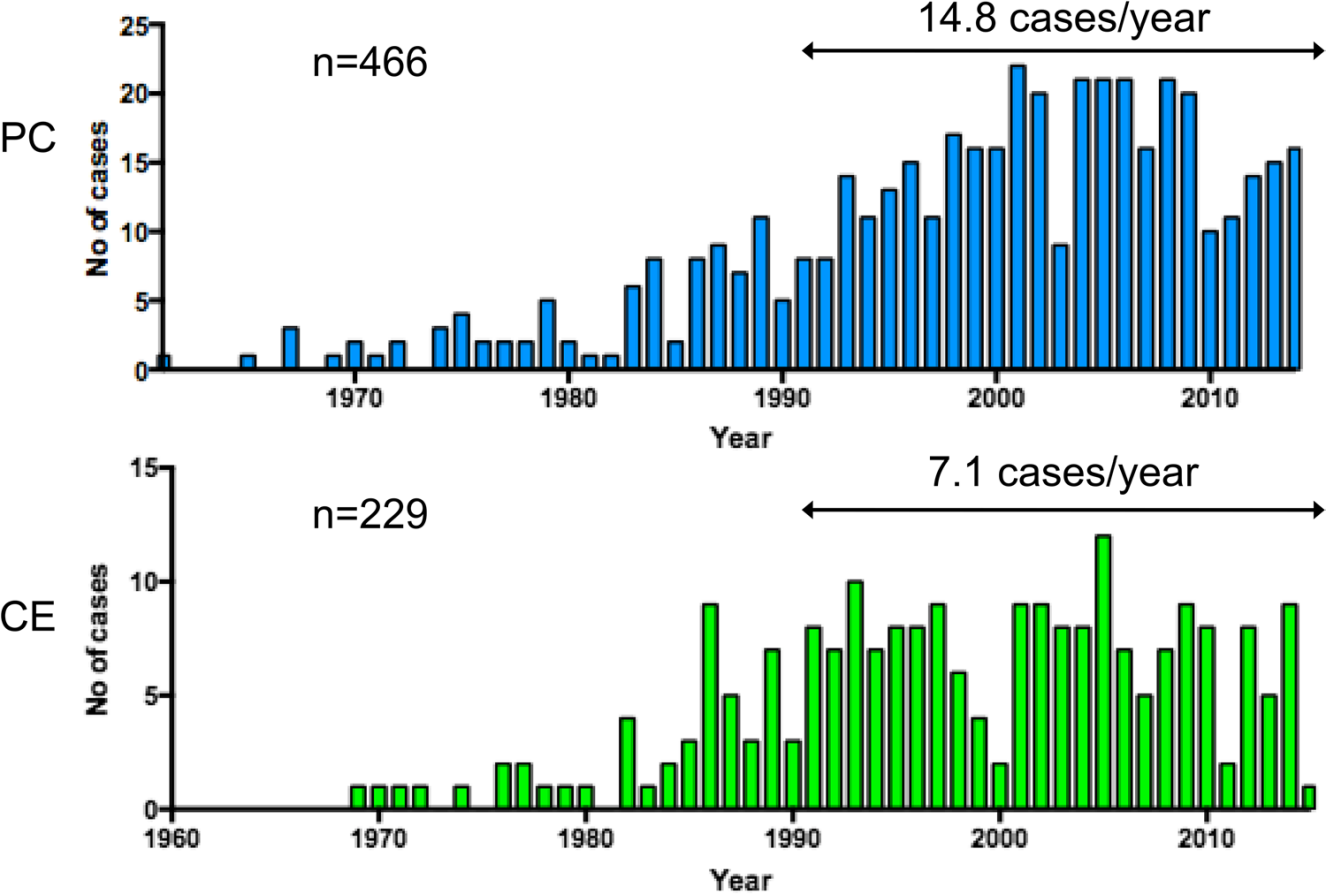
Annual occurrence of PC and CE in Japan. *PC* persistent cloaca, *CE* cloacal exstrophy

**Fig. 2:**
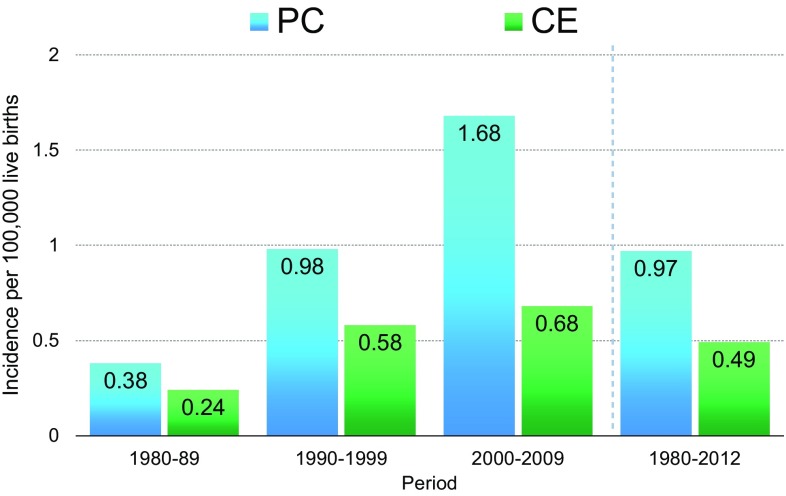
Prevalence of PC and CE patients per 100,000 live births for 1980–1989, 1990–1999, 2000–2009, and 1980–2012 in Japan. *PC* persistent cloaca, *CE* cloacal exstrophy

The oldest cases of PC and CE in this nationwide survey were found in 1961 and 1969, respectively (Fig. 1). In the past 25 years, from 1990 to 2014, the average annual occurrence was 14.8 and 7.1 cases for PC and CE, respectively. The overall prevalence of patients per 100,000 live births from 1980 to 2012 was 0.97 and 0.49 for PC and CE, respectively. The incidence of PC has been increasing over the past 30 years while that of CE has remained relatively stable (Fig. 2).

Prior to 1960, nearly all CE patients died due to intestinal obstruction and urosepsis [6]. The prevalence of PC per 100,000 live births was reported to be 2.0 in one study [7], while the prevalence of CE per 100,000 live births in several studies was 0.4 [8], 0.5 [9], or 0.6 [10], roughly corresponding to 1 in every 170,000 and 250,000 live births. An International Clearinghouse for Birth Defects Surveillance and Research study found that the prevalence of CE per 100,000 births was 0.76, which was an average of 18 surveillance programs, with a range of 0.6–2.25 [11]. These findings suggest that the prevalence of PC and CE in Japan might be similar to those of Western countries.

#### Antenatal detection of fetal anomalies

**Fig. 3:**
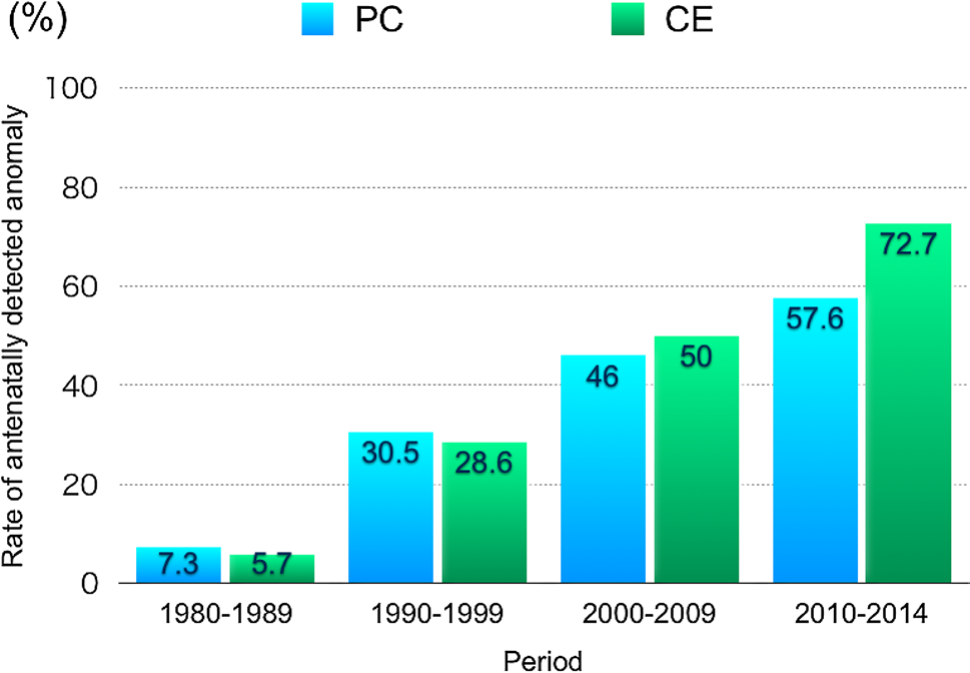
Trends in the rate of antenatal detection of fetal anomalies in PC and CE patients. *PC* persistent cloaca, *CE* cloacal exstrophy

The incidence of antenatal detection of fetal anomalies in PC and CE patients has been steadily increasing in both PC and CE patients in Japan (Fig. 3). In PC patients, pelvic cyst, hydronephrosis, oligohydramnios, and ascites were frequently found by ultrasonography. The signs for PC patients are mainly those of abnormalities inside the body, while for CE patients, the signs are mainly visible on the body surface, such as omphalocele, myelomenigocele, and abnormal genitalia, all of which are detected by ultrasonography.

Even though the antenatally detectable abnormalities in PC patients are mainly those of associated anomalies, hydronephrosis and bilobed or multilocular pelvic cyst in female fetuses have been suggested to be signs of PC itself [12, 13]. The rate of diagnosis of PC has been reported to be as low as 6%, even though the rate of detection of fetal anomalies in PC patients was 54% [14]. An accurate antenatal diagnosis of CE was achieved with fetal magnetic resonance imaging (MRI), which revealed a protuberant abdominopelvic contour, absence of a normal bladder, and a meconium-filled rectum and colon [15]. Serial MRI studies have also been suggested to be useful in the accurate diagnosis and understanding of the associated pathologies in the affected fetus [16].

#### Mode of delivery

In our survey, the rates of vaginal delivery were 54.8 and 51.7% in mothers of PC and CE fetuses, respectively. The rates of cesarean section in mothers of PC and CE fetuses were also similar, with rate of 37.7 and 42.0%, respectively. However, the main reason for a cesarean section was different between mothers of PC and CE fetuses. In mothers of PC fetuses, fetal distress was the most frequent reason for cesarean sections (31.7%), followed by fetal diseases (24.4%) and fetopelvic disproportion (23.7%). On the contrary, in mothers of CE fetuses, fetal disease (23.7%), myelomeningocele (15.8%), threatened premature delivery (15.8%), and early rupture of membranes (15.8%) were the main reasons for cesarean section.

PC is not considered an indication for cesarean section. However, in severe forms of abdominal wall defect, such as cloacal exstrophy, multi-disciplinary counseling and management are recommended [17]. In CE, there are complex management issues, such as gender assignment, a treatment plan and assessment of disability, and optimal management that require collaboration among specialists.

The median number of gestational weeks at delivery of the patients was significantly longer in PC patients than in CE patients (38.0 vs. 36.0 weeks, *p* < 0.0001). Correspondingly, PC babies were significantly heavier than CE babies (2,732 vs. 2441 g, *p* < 0001).

#### Associated anomalies

**Fig. 4:**
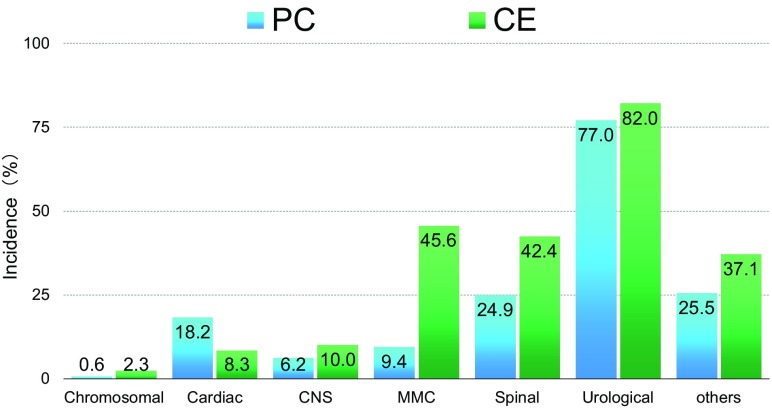
Associated anomalies in PC and CE patients. *PC* persistent cloaca, *CE* cloacal exstrophy, *Chromosomal* chromosomal anomaly, *Cardiac* cardiac anomaly, *CNS* anomaly of central nervous system, *MMC* myelomeningocele, *Spinal* spinal anomaly, *Urological* anomaly of urinary tract

The associated anomalies are summarized in Fig. 4. Chromosomal anomalies were rare in both PC and CE patients, but cardiac anomaly was much more frequently observed in PC patients than in CE patients. Of note, myelomeningocele and other spinal anomalies were found in 45.6 and 42.4% of CE patients, respectively. As a result, about 90% of CE patients had some type of anomaly of the spinal tract at birth. The association of spinal anomalies might be an important factor influencing the diminished bowel and bladder function in CE patients. Urinary tract anomalies were equally observed in both groups of patients at a frequency of roughly 80%. The urinary tract anomalies are shown in Table 2. Hydronephrosis was the most frequently found in both PC and CE patients, followed by a variety of diseases. The prevalence of renal hypoplasia or dysplasia, multicystic dysplastic kidney, and horseshoe kidney was higher in PC patients than in CE patients.

**Table 2:** Urinary tract anomalies in PC and CE patients

	PC cases (%)	CE cases (%)
Hydronephrosis	136 (29.1)	56 (24.5)
Hypoplasia or dysplasia	72 (15.5)	19 (8.3)
Renal agenesis	44 (9.4)	19 (8.3)
Megaureter	30 (6.4)	15 (6.6)
Duplex system	15 (3.2)	8 (3.5)
Multicystic DK	15 (3.2)	1 (0.4)
Horseshoe kidney	12 (2.6)	2 (0.9)
Ureterocele	3 (0.6)	2 (0.9)

*PC* persistent cloaca, *CE* cloacal exstrophy, *DK* dysplastic kidney

Regarding anomalies of the internal genitalia in PC patients, a double uterus (50.0%) or double vagina (35.2%) was frequently observed. Retention of fluid, such as hydrocolpos (23.6%), hydrometra (23.6%), and hydrosalpinx (5.4%) was the next most frequent after duplex anomalies. In total, 90% of patients had some type of anomaly of the internal genitalia. In CE patients, the presence of a uterus, vagina, fallopian tube, and testes was confirmed in 25.0, 12.7, 20.0 and 26.2% of patients, respectively.

In a single-center study of 17 CE patients [18], abnormalities of the vertebral column and/or spinal cord were found in 16 patients (94.1%). Four patients had lipomyelomeningocele, and nine had lipomeningocele. Another single-center analysis of 25 CE patients [19] found vertebral anomalies in all 25 patients. In addition, all 19 patients who underwent MRI showed myelodysplasia. Of these 19 patients, 15 had myelocystocele, 2 had lipomeningocele, and 2 had meningocele. The findings from these two studies are quite in accordance with those of the present survey that 90% of the patients were associated with myelomeningocele and/or spinal anomalies. Regarding the association of urological anomalies in PC patients, 32 (68.0%) out of 47 patients were reported to have urological defects [20].

Regarding the reproductive system, vaginal agenesis, double vagina, and single vagina were reported in 12 (11.4%), 42 (40.0%), and 52 (49.5%) of 105 PC patients in single-center study, respectively [21]. Pena [20] described a wide spectrum of the anatomic variation in PC patients, where different degrees of vaginal and uterine separation was found in half of 50 patients. In genetically female CE patients, two widely separated vaginas were confirmed when the patients underwent vaginal reconstruction at puberty [6, 22]. The ovaries and tubes are generally normal in female PC patients [23]. In genetically male CE patients, the phallus is also divided and separated. Husman et al. [24] reported diminutive or absent penis in 30% of patients. Even though cryptorchidism was frequent in genetically male CE patients, the testicular histology was reported to be preserved [25].

#### Gender assignment

In this study, gender assignment in half of the CE patients was determined based on the results of a sex chromosome analysis. A total of 91 CE patients were thereby determined to be genetically male, and 116 were determined to be genetically female, resulting in a male-to-female ratio of 0.78. Of these 91 genetically male CE patients, 21 were raised as girls.

Other studies have reported a genetically male-to-genetically female ratio of 0.86 [19] and 1.27 [26]. Regarding the gender identity outcome, female-raised 46XY CE patients are reportedly at risk of later patient-initiated gender re-assignment to male after female assignment in infancy or early childhood, an outcome which has also been observed in patients with penile agenesis or penile ablation [27]. In our series, four 46XY CE cases assigned as female changed their sex to male in adolescence.

### Surgical treatments shortly after birth

Stoma was created in most PC patients (95.5%) and CE patients (91.3%). In 104 CE patients (45.4%), a stoma was placed in the hindgut, while the ileum was used in 51 cases (22.3%). In contrast, the transverse or sigmoid colon was mainly used in 60.9% and 19.7% of PC patients, respectively. Vesical operation was performed in 25.1% of PC patients, while this rate was 80.8% in CE patients. In PC patients, vesicostomy was performed in 86.5% of the patients who underwent vesical operation, while primary closure of the bladder was performed in 69.1% of CE patients who underwent vesical operation. In CE patients, a variety of other operations were performed. Pubic and pelvic bone plasty and abdominal wall closure were performed in 64.2 and 80.8% of CE patients, respectively.

### Radical operations

#### Anoplasty and vaginoplasty

Table 3 shows the major operative procedures for anoplasty without vaginoplasty and number of patients who underwent the corresponding operations. Anoplasty without vaginoplasty was performed in 32.8% of PC patients and 7.9% of CE patients, and abdomino-perineal pull-through operation was the most frequent method in both groups of patients, followed by posterior sagittal anorectoplasty (PSARP) and sacroperineal operation.

**Table 3:** Anoplasty without vaginoplasty in PC and CE patients

Method of operation	PC 153 cases	CE 18 cases
Abdomino-perineal pull-through	54	8
PSARP	41	2
Sacroperineal pull-through	14	2
Abdomino-sacroperineal pull-through	11	
Laparoscopic operation	8	
Perineal approach	3	

*PC* persistent cloaca, *CE* cloacal exstrophy, *PSARP* posterior sagittal anorectoplasty

Table 4 describes the major operative procedures for anoplasty and vaginoplasty performed simultaneously, which was exclusively found in PC patients. Cases in which the procedures for the vaginoplasty and anoplasty were the same or different are shown separately. Posterior sagittal anorectourethrovaginoplasty (PSARUVP) was the method most frequently used for anovaginoplasty, while total urogenital mobilization and intestinal interposition was the method most frequently used for vaginoplasty. In contrast, in CE patients, only 24 patients (10.5%) underwent vaginoplasty. The main type of operation was reconstruction using the ileum, bladder, colon, or cecum. The rate of patients who decided to have permanent stoma was 10 times higher in CE patients than in PC patients (73.8 vs. 7.3%). This difference might be due to the higher incidence of myelomeningocele in CE patients than in PC patients.

**Table 4:** Anoplasty with vaginoplasty in PC patients

Anovaginoplasty in the same operation method	232 cases	Vaginoplasty performed by the different method to anoplasty	144 cases
PSARUVP	170	TUM	41
Abdomino-perineal pull-through	6	Intestinal interposition	35
TUM	4	Anterior skin flap	24
Perineal anovaginoplasty	4	PUM + skin flap	9
Hendren’s operation	2	Skin flap	9
PUM	1	Vaginal switch	5
Sacroperineal pull-through	1	PUM	2

*PC* persistent cloaca, *PSARUVP* posterior sagittal anorectourethrovaginoplasty, *TUM* total urogenital mobilization, *PUM* partial urogenital mobilization

#### Operation on the urinary tract and outcomes

Bladder augmentation was performed in 7 PC patients (1.5%) and 62 CE patients (27.0%), respectively. Route creation for clean intermittent catheterization (CIC) was done in 70 PC patients (15.0%) and 16 CE patients (7.0%). The rate of a radical operation for VUR was similar between PC and CE patients (15.0 vs. 15.3%). Among CE patients, orchidopexy was performed in 37 patients (16.1%) and phalloplasty in 17 (7.4%).

Regarding the renal function, 35 PC patients (7.5%) and 26 CE patients (11.4%) had a creatinine level above 1.0 mg/ml. Even though the renal function was relatively well preserved, bladder dysfunction was observed in 152 PC patients (32.6%) and 139 CE patients (60.7%). The rate of patients with CIC was similar between the PC (105 cases; 22.5%) and CE patients (65 cases; 28.4%). The number of cases starting dialysis or receiving kidney transplantation was 15 PC cases and 3 CE cases.

#### Reproductive organ function

Menstruation was observed in 178 PC patients (38.4%) and 45 CE patients with a karyotype of 46XX (38.8%). The occurrence of a menstrual disorder, menstruation blood outflow block, volume disorder, and cycle disorder was observed in 35.4, 22.5, 31.4, and 42.1% of the 178 PC patients and in 58.7, 48.9, 42.2, and 30.4% of the 45 CE patients, respectively.

#### Literature review of radical operations

Surgical repair of the anus, bladder, and vagina in CE patients is still challenging. The high prevalence of neurological abnormalities might affect the post-operative urinary and bowel continence, thereby limiting the indications of anoplasty. In a study of the neurological function of 21 CE patients [19], 15 (71.4%) were community ambulators, while 1 (4.8%) was a nonfunctional ambulator and 6 (28.6%) were nonambulators. Therefore, roughly one third of CE patients have some degree of motor dysfunction of the lower extremities. In our series, half of CE patients were associated with myelomeningocele, which might be related to the low incidence of radical operation in CE patients. In a large series of 50 CE patients [26], 40 underwent extensive reconstructive surgery. Of these 40 patients, the number who underwent bladder augmentation, pull-through of the colon, and vaginal reconstruction was 35 (87.5%), 25 (14.3%), and 32 patients (80%), respectively. A total of 31 of these 40 reconstructed cases were dry, and acceptable bowel continence was achieved in 19 of 25 pull-throughs. The hind gut is strongly recommended for the initial colostomy, since future colon pull-through is essential for successful bowel management [28, 29]. Therefore, the use of the colon for urologic and genital reconstruction should be avoided.

In a large series of PC patients consisting of 490 cases [30], the length of the common channel affected the surgical outcomes. Patients with a short common channel (length <3 cm) comprised 56% of patients and showed a better bowel habit and urinary continence after radical operation than those with longer channels. In these patients, radical operation was performed via the posterior sagittal approach, and total urogenital mobilization without laparotomy. In this study, the method chosen for anoplasty without vaginoplasty was either abdomino-perineal pull-through or PSARP. In addition, while PSARP was the most frequently used method for anovaginoplasty where both operations were performed via the same method, total urogenital mobilization or intestinal interposition was the most frequently used when vaginoplasty was performed via a different method from anoplasty.

### Social and school life

Patients with lovers or spouses comprised 5.8 and 2.2% of the PC and CE patients, respectively. Coitus before marriage was reported in about 3.0 and 2.6% of PC and CE patients. Seventeen PC patients (3.6%) and 5 CE patients (2.2%) were married. Childbearing was noted in four PC patients and none of the CE patients.

The rate of patients who attended a special needs school was higher among CE patients than PC patients (14 CE patients [6.1%] vs. 9 PC patients [1.9%]). Bowel and urinary troubles at school were noted in 67 (28.1%) and 59 (25.2%) PC patients and 28 (28.5%) and 42 (42.4%) CE patients, respectively. While the rate of bowel trouble was similar between the two groups of patients, urinary trouble was much more frequent in CE patients than in PC patients.

Childbirth is sporadically reported among both PC patients [31, 32] and CE patients [33]. One PC case delivered vaginally in the first pregnancy, and then cesarean section was performed for a triplets pregnancy after in vitro fertilization [31, 32]. The risks associated with cesarean section due to multiple previous operations have been well addressed. However, careful consideration of the patient’s genital condition and well-organized team counseling might be advisable before childbirth is attempted in PC and CE patients.

The quality of life among PC and CE patients is an important clinical issue, as many patients are now living longer due to the advancements of modern medicine. One study at Johns Hopkins University [34] suggested that a reduction in incontinent stomas, assistance with ambulation, and improved cosmesis might be useful for improving these patients’ quality of life. CE patients are reported to have relatively good psychological functioning, and appropriate support for these patients can ensure they have remarkably well-adjusted lives [35].

## Conclusions

A comparative study of PC and CE patients may be useful for clarifying the characteristics of both diseases. In Japan, the prevalence of CE patients was just half of that of PC patients, and the major anomalies differ between these two groups of patients. We also clearly showed that the surgical outcomes of PC and CE patients were still not satisfactory. A majority of patients require continual care from childhood to adulthood, especially in the treatment of urological and gynecological morbidities. Therefore, treatment guidelines for the smooth transitional care of PC and CE patients should be established, a task currently being attempted by our research team.
